# A Large-Scale Outbreak of Echovirus 30 in Gansu Province of China in 2015 and Its Phylodynamic Characterization

**DOI:** 10.3389/fmicb.2020.01137

**Published:** 2020-06-10

**Authors:** Jianhua Chen, Zhenzhi Han, Haizhuo Wu, Wenbo Xu, Deshan Yu, Yong Zhang

**Affiliations:** ^1^Key Laboratory of Infectious Diseases in Gansu Province, Gansu Center for Disease Control and Prevention, Lanzhou, China; ^2^WHO WPRO Regional Polio Reference Laboratory and National Health Commission Key Laboratory of Biosafety, National Institute for Viral Disease Control and Prevention, Chinese Center for Disease Control and Prevention, Beijing, China; ^3^Center for Biosafety Mega-Science, Chinese Academy of Sciences, Wuhan, China

**Keywords:** echovirus 30 (E-30), phylogenetic analysis, molecular epidemiology, phylodynamics, encephalitis

## Abstract

**Background:**

Echovirus 30 (E-30) has been investigated and reported worldwide and is closely associated with several infectious diseases, including encephalitis; myocarditis; and hand, foot, and mouth disease. Although many E-30 outbreaks associated with encephalitis have been reported around the world, it was not reported in northwest China until 2015.

**Methods:**

The clinical samples, including the feces, serum, throat swabs, and cerebrospinal fluid, were collected for this study and were analyzed for diagnosis. E-30 was isolated and processed according to the standard procedures. The epidemiological and phylogenetic analysis were performed to indicate the characteristics of E-30 outbreaks and phylodynamics of E-30 in China.

**Results:**

The E-30 outbreaks affected nine towns of Gansu Province in 2015, starting at a school of Nancha town and spreading to other towns within 1 month. The epidemiological features showed that children aged 6–15 years were more susceptible to E-30 infection. The genotypes B and C cocirculated in the world, whereas the latter dominated the circulation of E-30 in China. The genome sequences of this outbreak present 99.3–100% similarity among these strains, indicating a genetic-linked aggregate outbreak of E-30 in this study. Two larger genetic diversity expansions and three small fluctuations of E-30 were observed from 1987 to 2016 in China, which revealed the oscillating patterns of E-30 in China. In addition, the coastal provinces of China, such as Zhejiang, Fujian, and Shandong, were initially infected, followed by other parts of the country. The E-30 strains isolated from mainland of China may have originated from Taiwan of China in the last century.

**Conclusion:**

The highly similar E-30 genomes in this outbreak showed an aggregate outbreak of E-30, with nine towns affected. Our results suggested that, although the genetic diversity of E-30 oscillates, the dominant lineages of E-30 in China has complex genetic transmission. The coastal provinces played an important role in E-30 spread, which implied further development of effective countermeasures. This study provides a further insight into the E-30 outbreak and transmission and illustrates the importance of valuable surveillance in the future.

## Introduction

Enteroviruses belong to the genus *Enterovirus* of the *Picornaviridae* family, which now consists of 15 species designated as *Enterovirus* A–L and *Rhinovirus* A–C (Zell et al., 2017). *Enterovirus* A–D (EV-A, EV-B, EV-C, and EV-D) are closely associated with human diseases and were studied for the purpose of public health. The genomic length of enterovirus is ∼7.5 kb, which contained a long open reading frame (ORF), with 5′-untranslated region (UTR) and 3′-UTR. The polyprotein could be cleaved into *P1*, *P2*, and *P3* protein precursors, followed by the further cleavage into *VP1–VP4*, *2A–2C*, and *3A–3D*, respectively. The *VP1* coding region of enterovirus contained the major antigenic neutralization sites responsible to immunoreaction. The molecular typing approach, which primarily based on the *VP1* coding region of enterovirus, was accepted as the major method for serotyping (Rohll et al., 1995; Bergamini et al., 2000). With the development of molecular typing technologies, there are more and more new serotypes of enteroviruses have been found, with the *VP1* coding region and partial *VP1* coding region targeted (Oberste et al., 2005; Yozwiak et al., 2010; Tokarz et al., 2013; Zhang Y. et al., 2014; Han et al., 2018). This method has become the gold standard for enterovirus typing and detection and allows for greater convenience than serotyping (Oberste et al., 1999a, b). EV-B consists of 63 serotypes, including coxsackievirus (CV)-A9, CV-B group (serotypes 1–6), echovirus (serotypes 1–7, 9, 11–21, 24–27, 29–33), and newly identified enteroviruses (serotypes 69, 73–75, 77–88, 93, 97–98, 100–101, 106–107, 110–113) (Adams et al., 2017). These serotypes of enteroviruses are usually associated with a series of diseases, such as encephalitis; myocarditis; hand, foot, and mouth disease (HFMD); acute flaccid paralysis (AFP); and other diseases. For example, CV-B3 has been linked to myocarditis and encephalitis, whereas it could also cause outbreaks of HFMD as reported recently (Tao et al., 2012; Wu et al., 2013; Han et al., 2019). CV-B5 is an important pathogen that caused the outbreaks of encephalitis and HFMD (Han et al., 2012; Chen et al., 2013).

Echovirus 30 (E-30), which was classified as a member of *Enterovirus* species B, has caused several diseases, including encephalitis, HFMD, myocarditis, and other mild clinical manifestations, such as fever and headache (Tapparel et al., 2013; Adams et al., 2017; Rasti et al., 2019). Several E-30 outbreaks in the world have been reported and analyzed to confirm the epidemiological characteristics and evolutionary patterns, which have provided valuable information for disease control (Milia et al., 2013; Wieczorek et al., 2016; Chen et al., 2017). The first molecular epidemiological study of E-30 was reported in 1999, which revealed the temporal dynamics and genetic diversity in the world, whereas the prototype was sampled in 1958 (Oberste et al., 1999b; Palacios et al., 2002). There were several reports of encephalitis caused by E-30 worldwide (Wang et al., 2002; Cabrerizo et al., 2008; dos Santos et al., 2011; Kim et al., 2012; Milia et al., 2013), and the E-30 isolates were frequently detected from cases of encephalitis, indicating epidemiological relationship between E-30 and encephalitis. In rare cases, it can cause cute myalgia, rhabdomyolysis, and acute flaccid paralysis in immunosuppressed transplant recipients (Mauri et al., 2019; Sousa et al., 2019).

In 2001, an outbreak of encephalitis occurred in Taiwan, China. One thousand one hundred thirty cases of enterovirus infection were reported, and the detection rate of E-30 was 16.6% (Wang et al., 2002). In Jiangsu Province of China in 2003, 1,681 patients with encephalitis were reported, and E-30 was confirmed as the etiologic agent of this outbreak (Zhao et al., 2005). Similar incidences of E-30 outbreaks were reported in Shandong Province of China, showing that E-30 was a major pathogen in encephalitis patients (Tao et al., 2014). The outbreaks of E-30, which were closely associated with encephalitis, were also frequently reported in other parts of the world, including Italy, Bulgaria, France, United Kingdom, Spain, Brazil, and Korea (Cabrerizo et al., 2008; Yang et al., 2013; Mladenova et al., 2014; Holmes et al., 2016). Surprisingly, a recent report showed that E-30 was detected in an outbreak of acute myalgia and rhabdomyolysis, indicating that E-30 possibly caused severe non-neuropathic diseases and that E-30 could cause a variety of clinical symptoms (Sousa et al., 2019).

In this study, we have described a large-scale outbreak of E-30 associated with viral meningitis, fever, headache, nausea, and vomiting symptoms, which led to aggregated cases in Gansu province of China in 2015. The clinical features, molecular epidemiological characteristics, and evolutionary dynamics of this outbreak were analyzed. Furthermore, the detailed transmission patterns were tracked to illustrate the prevalence scope and extent of occurrence of E-30 in China. This is the first report about encephalitis caused by E-30 in the northwest region of China. The investigation provides further insight into the epidemiological patterns and evolutionary history of E-30 in China.

## Materials and Methods

### Case Definition and Investigation

**FIGURE 1:**
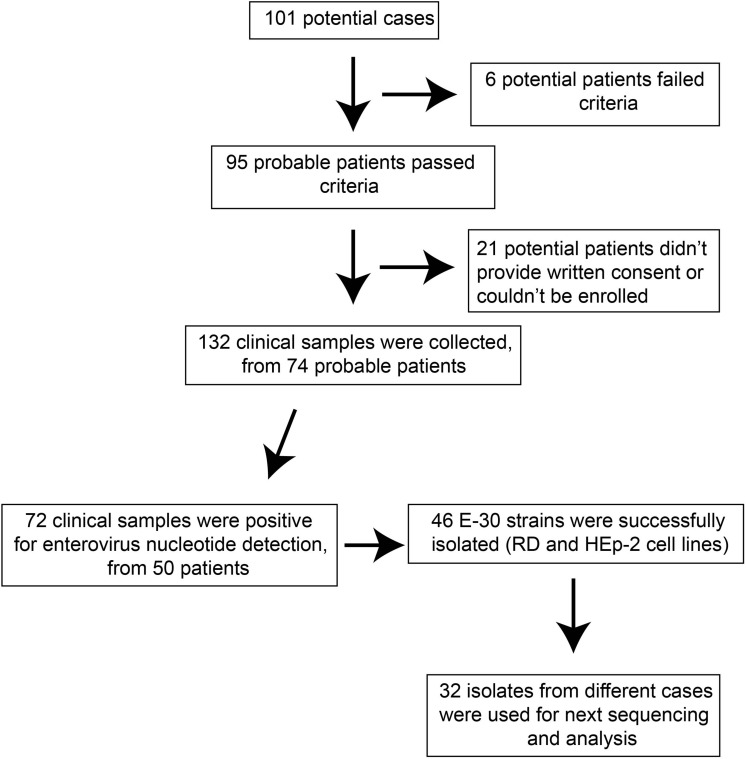
The overview of flow presented in this study.

The probable cases were reported, and samples of patients were referred to the laboratories for pathogen detection. The local Centers for Disease Control and Prevention (CDC) staffs collected the clinical samples in several local hospitals that have patients with viral encephalitis; the use of their clinical samples was explained to the guardians of children, and written consent was signed by guardians of children for permitting analysis of their clinical samples. Patients were classified as having viral encephalitis infection in Gansu province between June 27 and August 5 of 2015 if they had encephalitis, high fever (≥38°C), nausea, vomit, and other neurological symptoms, such as seizures, unconsciousness, and autonomic nervous system dysregulation (Figure 1). Following this outbreak, 101 suspected cases were recognized by HFMD surveillance system. A total of 95 patients met the clinical diagnostic criteria of encephalitis according to their clinical manifestations (Table 1). The representative clinical samples from the 74 probable patients were collected for lab detection (Figure 1). We have excluded the common infections of Japanese encephalitis virus, adenovirus, mumps virus, and human herpesvirus hominis using ELISA immunoglobulin M (IgM) method and real-time quantitative reverse transcription polymerase chain reaction (qRT-PCR).

**TABLE 1:** The demographic characteristics of probable cases and laboratory-confirmed cases from the encephalitis outbreak in Gansu, 2015.

	Probable cases	Laboratory-confirmed cases
**Age group (*n*,%)**		
≦5	10 (10.5%)	9 (18%)
6–10	49 (51.6%)	22 (44%)
11–15	27 (28.4%)	12 (24%)
≥16	9 (9.5%)	7 (14%)
**Symptoms (*n*,%)**		
High fever (≥ 38°C)	95 (100%)	48 (96%)
Headache	28 (29.5%)	12 (24%)
Nausea and vomit	58 (61.1%)	34 (68%)
Neurological symptoms (listlessness and unconsciousness)	59 (62.1%)	30 (60%)
**Sex (*n*,%)**		
Male	62 (65.3%)	34 (68%)
Female	33 (34.7%)	16 (32%)
**Region (town)**		
Nancha	43 (45.3%)	21 (42%)
Guangzhi	24 (25.3%)	17 (34%)
Other	28 (29.5%)	12 (24%)
**Total**	95	50

### Sample Collection and Viral Isolation

For the purpose of public health under the investigation of the National Health Commission of the People’s Republic of China, we collected the feces (*n* = 27, 20.5%), serum (*n* = 66, 50%), throat swab (*n* = 25, 18.9%), and cerebrospinal fluid (*n* = 14, 10.6%). Real-time RT-PCR method was used to directly detect the enterovirus genome in clinical samples, with public probe and primers used (Cui et al., 2013; Zhang S. et al., 2014). We defined a laboratory-confirmed patient with laboratory evidence of enteroviruses infection. The positive samples were cultured and processed according to the standard procedures described before and were then inoculated into human rhabdomyosarcoma (RD) cells and human laryngeal epidermoid carcinoma (HEp-2) cells, provided by the WHO Global Poliovirus Specialized Laboratory for viral isolation. Infected cell cultures were harvested once complete EV-like cytopathic effect (CPE) was observed. All experimental protocols were carried out in accordance with the approved guidelines by WHO, as were reported before (Yan et al., 2010; Zhang et al., 2010b; Burns et al., 2013; Luo et al., 2013). As a result, 46 E-30 strains were harvested, with complete EV-like CPE. Subsequently, we randomly selected 32 isolates sampled from different patients for sequencing and phylogenetic analysis (Figure 1).

### Sequencing and Molecular Typing

Viral RNA was extracted from the cell cultures using the QIAamp Viral RNA Mini Kit (Qiagen, Germany). Reverse transcription PCR was performed to amplify the entire *VP1* coding region using the PrimeScript One-Step RT-PCR Kit, Ver.2 (TakaraBio, Dalian, China) using primers 490–493 as described before (Oberste et al., 2006). The entire *VP1* region of the amplicons was sequenced using ABI 3130 Genetic Analyzer (Applied Biosystems, Foster City, CA, United States). Using the BLAST server and the EV Genotyping Tool, the *VP1* sequences were analyzed to determine the serotypes (Kroneman et al., 2011). The genome sequence of the entire *VP1* region has been deposited in the GenBank database under accession number MN590241–MN590272.

### Phylogenetic Analysis

For molecular epidemiology study of E-30, the entire *VP1* sequences of E-30 from GenBank database were downloaded (∼1,400 sequences available until April 2019). First, based on the entire VP1 coding region of E-30, we constructed the maximum likelihood phylogenetic tree and randomly selected the representative strains of E-30 from each branch of phylogenetic tree. In addition, the representative sequences should cover more regions to obtain higher geographical representation and cover each cluster of phylogenetic trees for obtaining higher phylogenetic representation. Second, the datasets were filtered by discarding the low-quality sequences and incomplete genome sequences. At last, we randomly selected 36 representative entire *VP1* sequences for genotyping, including an E-21 sequence as an outgroup. To assess the mean genetic distance among different genotypes, we calculated the mean genetic distance between genotypes and within each genotype using the Kimura two-parameter model.

In general, 25% of the genome difference of enterovirus is used as the criteria for serotyping, while 15% of the genome difference is used as the criteria for genotyping; this is widely accepted for serotyping and genotyping of enterovirus, such as EV-A71, CV-A16, and CV-A6 (Brown et al., 1999; Oberste et al., 1999a, b; Mizuta et al., 2005; Zhang et al., 2010a, 2013; Song et al., 2017). We refer to the published genotyping criteria of EV-A71, CV-A16, and CV-A6 and combined with the latest research progress of E-30 (Lema et al., 2019; Maruo et al., 2019; Mauri et al., 2019; Sousa et al., 2019). In addition to being able to better represent the E-30 sequences in the world, we also avoid the redundancy sequences of E-30 for genotyping, which leads to the confusion and divergence of enterovirus genotyping.

A total of 446 entire *VP1* genome sequences acquired from China (until April 2019), which were extracted from ∼1,400 entire *VP1* genome sequence pools with known sampling dates and locations in the world, were used to analyze the phylodynamic in China (Supplementary Table S1). Genome sequences were aligned using the Muscle method implemented in MEGA software (version 7.0) (Kumar et al., 2016). The topology of maximum likelihood phylogenetic tree was assessed using the RaxML software (version 8.0) (Stamatakis, 2014).

Using Bayesian inference method implemented in BEAST software (version 1.8.4), the maximum clade credibility (MCC) tree and the coalescent-based Gaussian Markov random field (GMRF) skyride plots were inferred, with the genome substitution model of GTR + I + Γ supported by the ModelGenerator (version 0.85) (Drummond et al., 2012). The randomization tests in R package (version 3.5.0) using the TipDatingBeast package were performed to determine the temporal signal in the data (Rieux and Khatchikian, 2017). Sufficient temporal signals of datasets are confirmed when 95% credibility intervals of rate estimation of real datasets do not fall into the 95% credibility intervals of rate estimation from date randomized replicates (Supplementary Figure S1). Sampling times of the sequences available were used to calibrate the molecular clock. A total of 15 dataset analyses was run combined with one genome substitution model, three different clock models, and five different Coalescent tree priors. The path sampling (PS) and stepping stone sampling (SS) analysis, which showed the marginal likelihood estimation results, were implemented in BEAST to choose the best parameters of Bayesian phylogenetic models (Supplementary Figure S2) (Baele et al., 2012). We checked the convergence and effective sample size (>200) of the parameters with Tracer software (version 1.7) (Rambaut et al., 2018). The output trees were summarized using the maximum clade credibility (MCC) topology from TreeAnnotator software (version 1.8.4), with a burn-in of the first 10% of sampled trees. To assess demographic dynamics of E-30 in mainland of China, the GMRF method with time-aware smoothing parameter was used to investigate the past population dynamics. The GMRF skyride plots were summarized and visualized using Tracer software (version 1.7.1) (Rambaut et al., 2018). The ggtree (version 1.16.3) was used to manipulate the phylogenetic tree for best performance (Yu et al., 2017).

### Ethics Statement

Written informed consent for the use of their clinical samples was obtained from all individuals or their guardians included in the study. The study was also supported by the Second Ethics Review Committee of the National Institute for Viral Diseases Control and Prevention, Chinese Center for Diseases Control and Prevention.

## Results

### Demographic and Clinical Characteristics of Large-Scale Outbreak Associated With E-30

A large-scale encephalitis outbreak caused by E-30 was confirmed through clinical characteristics, epidemiological evidences, and laboratory diagnosis. We defined the potential probable patients (*n* = 101), probable cases (*n* = 95), and laboratory-confirmed cases (*n* = 50) during epidemiological investigation (Figure 1 and Table 1). Among the probable cases (*n* = 95), male outnumbered female patients, with the ratio of male to female being 188% (Table 1). All cases had high fever (≥38°C), and headache accounted for 29.5% of all suspected cases. Of all the probable cases (*n* = 95), 61.1 and 62.1% had nausea, vomiting, and neurological appearance (listlessness and unconsciousness), respectively. Of the total number of probable cases (*n* = 95), 10.5% were children aged ≤5. Children aged 6–10 years accounted for the highest percentage at 51.6%, followed by children 11–15 years of age (28.4%). Children aged ≥16 only accounted for 9.5%. Several probable cases (*n* = 82, 86.3%) were school students, while other 13 cases were from different professions, including farmers (Table 1 not shown). From the geographical distribution angle, the Nancha town and Guangzhi town constituted 45.3 and 25.3% within the probable cases (*n* = 95), respectively, whereas seven other towns only accounted for 29.5% within the probable cases. A total of 60% (9/15) towns of this prefecture were attacked by the large-scale infectious disease outbreak.

**FIGURE 2:**
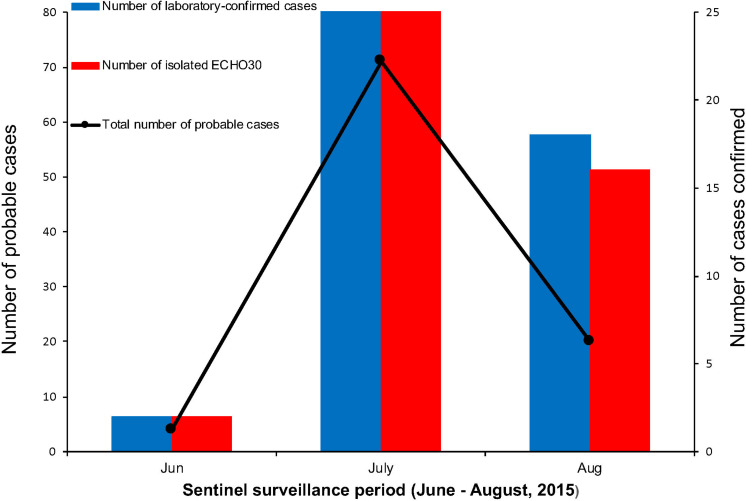
The number of probable and laboratory-confirmed cases, as well as the isolated E-30 strains reported. The line chart shows the sentinel surveillance data of probable cases reported from June to August, 2015, corresponding to the left vertical coordinates. The bar chart represent the laboratory-confirmed cases and isolated E-30 strains corresponding to the right vertical coordinates.

Among the laboratory-confirmed cases (*n* 50), the male outnumbered female patients, with a proportion of 210% (Figure 1 and Table 1). The ratios of demographic characteristics between probable cases and lab-confirmed cases were similar (Table 1). The collective outbreak, which presented similar symptoms for all patients and showed rapid diffusion in a short timescale, exhibited the increasing transmission ability of E-30 (Figure 2). The major period of E-30 transmission, which caused the most infection and outbreak, is July. A total of 91 probable cases were reported in July and August, constituting 95.8% (91/95) of total probable cases (*n* = 95). It is consistent with the detected proportion of laboratory-confirmed cases and E-30 isolation. From the opening phase of E-30 transmission, E-30 spread to nine towns using only 1-month timescale, indicating the rapid transmission ability.

### The Detection and Isolation Results of E-30 Outbreak

In these clinical samples, 54.5% (72/132) were positive for EV genome detection (Table 2). The positive number of EV genome detection for feces, serum, throat swab, and cerebrospinal fluid are 22 (81.5%), 15 (22.7%), 24 (96%), and 11 (78.6%), respectively. We tried to inoculate RD and HEp-2 cell lines with all positive samples of enterovirus nucleic acid and showed different proportions of isolation of E-30 with total viral isolation ratio of 63.9% (46/72); 12 feces samples (44.4%), 0 serum samples (0%), 24 throat swabs (96%), and 10 cerebrospinal fluid samples (71.4%) were successfully used to isolate E-30 strains. About 10 randomly selected serum samples were subjected to ELISA IgM test, out of which 50% (5/10) serum samples tested positive for CV IgM.

**TABLE 2:** Genome detection and viral isolation results of the encephalitis outbreak in Gansu Province, 2015.

Species	Numbers	Positive number of EV	Number of isolated E-30*
Feces	27	22 (81.5%)	12 (44.4%)
Serum	66	15 (22.7%)	0 (0%)
Throat swab	25	24 (96%)	24 (96%)
Cerebrospinal fluid	14	11 (78.6%)	10 (71.4%)
Total	132	72 (54.5%)	46 (34.9%)

### The Genotype Identification of the E-30 Outbreak

**FIGURE 3:**
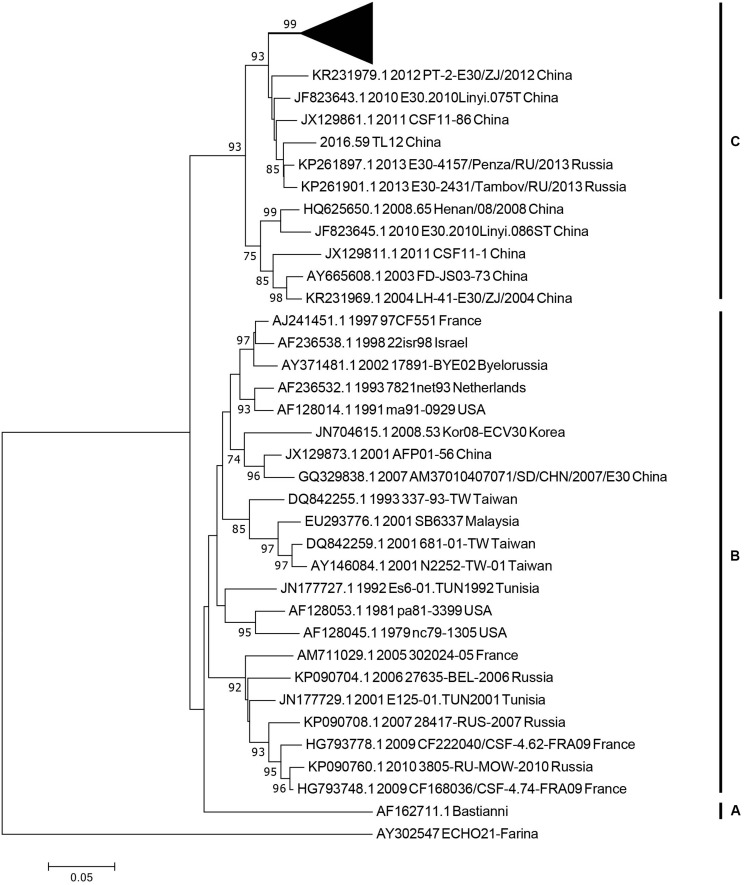
The phylogenetic tree based on the entire *VP1* genome of 36 representative strains isolated from 1958 (Bastianni strain of E-30 prototype) to 2016, with the E-21 strain used as an outgroup. The black triangle indicates the strains isolated from this study, and one strain was included for genotyping. The node numbers at each node show the bootstrap support constructed by neighbor-joining method with 1,000 bootstrap replicates. The letters on the right represent the genotypes of E-30, from genotypes A–C.

We used 36 entire *VP1* coding region sequences, including one E-21 genome sequence as an outgroup for genotyping (see section “Materials and Methods”). The neighbor-joining phylogenetic tree, based on the 36 entire *VP1* genome sequences with 1,000 bootstrap replicates, was constructed. According to the criteria of difference of at least 15% genetic distance over the entire *VP1* sequence, the genotype of E-30 are segregated into three distinct genotypes designated as genotype A, B, and C (Figure 3). The outgroup of E-21 strain and E-30 prototype strain isolated from America in 1958 formed a single genotype, respectively, and differed from other genotypes by 19.2–21.5%. The genotype B, which consisted of several strains isolated from many regions between 1979 and 2010, such as France, Netherlands, United States, Taiwan of China, and other regions, showed 16–21.5% genetic distance compared with other genotypes. The genotype C, which revealed the genetic differences between 16 and 19.2% among different genotypes, primarily was composed of Chinese isolates ranging from 2003 to 2016, besides two strains of E-30 from Russia. The mean genetic divergence within the genotype B and C showed 7 and 10.1%, respectively.

### Molecular Epidemiological Characteristics of E-30 in China

**FIGURE 4:**
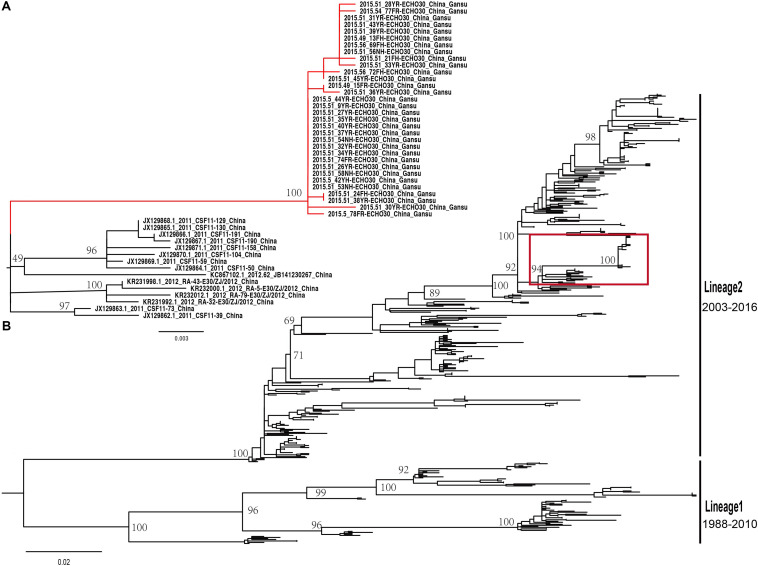
**(A)** The magnification based on the red box of **(B)** and the branches colored in red represent the E-30 isolates from the collective outbreak of this study in 2015. **(B)** The midpoint-rooted maximum likelihood phylogenetic tree of 446 E-30 strains isolated from China. The scale bars represent the substitutions per site per year. The lineages 1 and 2 show the transmission lineages of E-30 in China, including the timespan of E-30 strains.

The midpoint-rooted maximum likelihood phylogenetic tree showed that two lineages (lineage 1–2) circulated in China (Figure 4B). The mean genetic distance between the two lineages, which was calculated using the Kimura two-parameter model, is 18.1% and is larger than the mean genetic distance within these two lineages (7.3 and 9.6%), indicating the reliability of lineages division. The lineage 1, which circulated in the mainland of China and Taiwan of China earlier, consisted of fewer E-30 isolates recently and was infrequently detected in China. The strains of lineage 1 seemed to be disappearing in recent years, indicating that the E-30 evolved in nearly 20 years. However, the lineage 2, which circulated in China from 2003 till present, comprised mostly E-30 strains isolated from the mainland of China, including the encephalitis outbreak isolates of this study (Figure 4A, colored in red). The *VP1* genome sequences of these strains isolated from this study showed high similarity, with 99.3–100% identity among these *VP1* sequences. The close phylogenetic relationship, as shown in the maximum likelihood tree, also confirmed the occurrence of collective outbreak.

### Evolutionary Characteristics of E-30 in China

**FIGURE 5:**
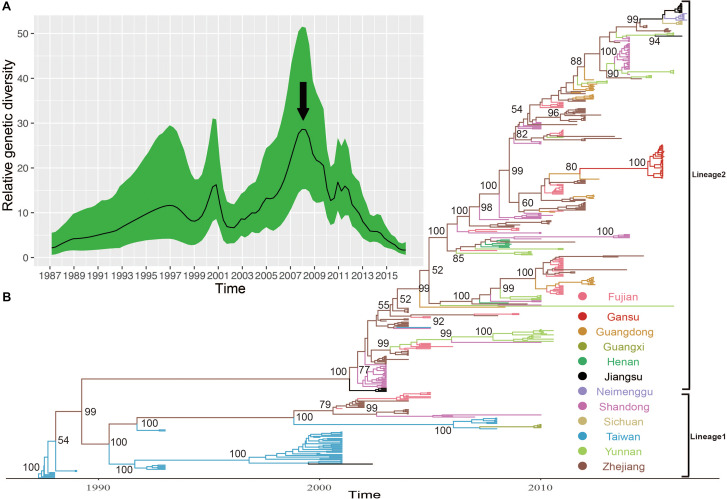
**(A)** The relative genetic diversity of E-30 sequences in China. The black arrows show the important timescale that the relative genetic diversity of E-30 decrease signally. **(B)** The maximum clade credibility (MCC) phylogenetic tree based on the entire *VP1* coding region in China and colored according to the locations of strains. The detailed information of isolates used in this study, including GenBank accession numbers, locations, and other essential information, are presented in Supplementary Table S1. The lineages 1 and 2 show the transmission lineages of E-30 in China, corresponding to Figure 4.

The MCC phylogenetic tree of 446 entire *VP1* coding region sequences of E-30, sampled from 1988 to 2016 in China, formed a ladder-like phylogenetic tree topology colored by locations of isolation. The MCC phylogenetic tree classified all E-30 strains into two lineages (lineage 1–2), consistent with the maximum likelihood phylogenetic tree described above (Figure 5B). The Bayesian phylogenetic analysis show that lineage 2 still dominated in China, whereas lineage 1 seemed to be disappearing. As shown in Figure 5, each clade did not locate in only one province, illustrating that E-30 transmitted and spread in several regions. Sporadic importing and exporting of cases among provinces of China were monitored, showing a certain level of genetic flow of E-30 among different regions. The clades of MCC phylogenetic tree, covering several provinces, showed a more complex distribution phenomenon compared with CV-B3 geographical distribution reported before (Han et al., 2019). For example, the strains isolated from Yunnan province almost located at each clade of MCC phylogenetic tree, indicating that the E-30 spread widely in China (Figure 5B, colored in light green).

The evolutionary dynamics of E-30 in China was estimated using a Bayesian method with relaxed molecular clock, including their evolutionary rate, tMRCA, and phylodynamic. The substitution rate for the *VP1* coding region of E-30 is 6.54 × 10^–3^ substitutions per site per year [95% highest posterior density (HPD), 5.76 × 10^–3^–7.33 × 10^–3^)], which is lower than the *VP1* evolutionary rate of EV-A71 and CV-A6 reported by others (McWilliam Leitch et al., 2012; Anh et al., 2018). However, the median value of substitutions rate of *VP1* sequences is slightly larger than that of CV-B3, indicating that the E-30 relatively evolved compared with CV-B3, whereas 95% HPD values of CV-B3 cover the threshold value of E-30 (Han et al., 2019). According to the estimated molecular clock, the common ancestor of lineage 1 was dated to about July of 1986 (95% HPD, November 1985–February 1987), whereas the strains of E-30 were isolated first in Taiwan of China in 1988. The isolates of lineage 2, which was estimated for tMRCA of April 2001 (95% HPD, June 2000–November 2001), emerged in Jiangsu and Shandong Province first in 2003. The results showed that E-30 had transmitted in China for approximately 2 years before it was detected in China.

Based on the *VP1* coding region, the coalescent reconstruction revealed the oscillating patterns on the genetic diversity of Chinese E-30 from 1996 onward (Figure 5A). The results showed that the genetic diversity of E-30 in China experienced two large expansions besides three small increases. In 2000 and 2008 nearby, the genetic diversity of E-30 arrived at the top and subsequently decreased dramatically. The increases of genetic diversity in 2000 were primarily associated with E-30 outbreaks in Taiwan of China, with 16.6% detection ratios of E-30 in 1130 enterovirus-infected patients with encephalitis (Wang et al., 2002). The E-30 strains isolated from Zhejiang, Fujian, Shandong, and Jiangsu Provinces closely clustered with the isolates from Taiwan of China (Zhao et al., 2005; Wang et al., 2006). With the decrease in relative genetic diversity of E-30 after 2008, it was coincided with the construction of hand, foot, and mouth pathogen surveillance net of China in 2008. The genetic diversity also showed a dynamic fluctuation between 2009 and 2012, especially the small-scale epidemics in 2011, revealing the transmission activities of E-30 among provinces. From 2012 till present, the population dynamics of E-30 showed weaker fluctuations over time, whereas it locally caused a large-scale outbreak in Gansu Province in 2015.

## Discussion

Although E-30 outbreaks were recorded in China, the deep evolutionary characteristics of E-30 remained unclear (Zhao et al., 2005; Tao et al., 2014; Chen et al., 2017, 2018). To assess the phylogenetic dynamic of E-30 in China and provide insight of E-30 evolution, we performed deep analysis for revealing the epidemiological features and transmission models. Our analysis revealed that this outbreak affected nine towns of the prefecture of Gansu Province and caused a severe infectious disease transmission. The Nancha town of Gansu Province has the most infected patients. The outbreak of E-30 started at a school in Nancha town in June and gradually spread to other towns in July, with a strong transmission ability. The cases reported presented common clinical symptoms, including high fever (≥38°C), nausea, vomiting, and neurological appearance (listlessness and unconsciousness), indicating a major clinical characteristics of E-30 surveillance. Males comprise higher ratio, with 65.3 and 68% cases in probable cases panel and laboratory-confirmed cases panel, respectively. Children aged between 0 and 15 accounted for 90.5 and 86% in probable cases panel and laboratory-confirmed cases panel, respectively. The results show that children are more easily attacked by E-30 infection, which is consistent with other enterovirus infections (Xing et al., 2014; Huang et al., 2018). However, the age group between 6 and 15 constituted the highest ratio. In the process of laboratory test, the throat swabs, cerebrospinal fluids, and feces presented high detection ratios, with the proportion of 96, 78.6, and 81.5%, respectively. However, the serum samples are not good samples for viral isolation compared with other clinical samples; therefore, when collecting clinical samples during E-30 outbreak, investigators do not collect serum for virus isolation.

The analysis of entire *VP1* sequences of chosen strains have shown that genotypes B and C have been cocirculating in the world and are responsible for many E-30 outbreaks (Wang et al., 2002; Kim et al., 2012; Yang et al., 2013; Maruo et al., 2019). Genotype C plays a leading role in the transmission of E-30 in China, including the strains of E-30 outbreak reported in this study. It is now evolving and spreading in different provinces of China. Two lineages (lineage 1–2) were formed in China, which have played an causal role in encephalitis outbreaks (Wang et al., 2002; Zhao et al., 2005; Yang et al., 2013; Tao et al., 2014; Chen et al., 2017). The strains of lineage 2 have been isolated from many provinces of China, such as Zhejiang, Guangdong, Shandong, Henan, Fujian, and Sichuan provinces. This lineage, diffusing in many provinces of China, evolved rapidly and caused several encephalitis outbreaks. For example, 1,681 encephalitis patients were reported in Jiangsu Province in 2003, and E-30 was confirmed as an etiologic agent of the outbreak (Zhao et al., 2005). It was also reported as a major pathogen of encephalitis cases in Shandong Province of China between 2006 and 2012 (Tao et al., 2014). From the maximum likelihood phylogenetic tree, spatial transmission events of E-30 were confirmed, and many sublineages cocirculated in several different regions of China. For example, the strains (GenBank accession number JX129864) isolated from Fujian Province cluster with the strains from Guangdong Province (GenBank accession number KC867102) (Figure 4A). This phenomenon was also observed in several other regions of China. The strain of 45R (GenBank accession number LC201508), which was isolated from Yunnan province in 2016, was encompassed by the strains (GenBank accession number KY048011-KY048043) isolated from Shandong Province. The 99.3–100% genome similarity based on the *VP1* genome of this study confirmed the occurrence of collective outbreak, although the strains were isolated from different towns of Gansu province.

Using the Bayesian phylogenetic inference method, the evolutionary dynamics of E-30 were analyzed. Sporadic importing and exporting of cases were observed in several provinces of China, indicating that E-30 transmitted through different regions and that the gene flow of E-30 among different provinces was apparent. The substitution rate of *VP1* coding region of E-30 is higher than that of CV-B3, indicating the relatively faster evolution extent of E-30 as compared to CV-B3 (Han et al., 2019). The low substitution rate, compared with EV-A71 and CV-A6, revealed relatively slower evolutionary levels, implying less possibility of outbreaks (McWilliam Leitch et al., 2012; Geoghegan et al., 2015; Anh et al., 2018). The timespan for the emergence of lineages 1 and 2 was 15 years, indicating chronic transmission and evolution. Two larger expansions of relative genetic diversity in China were observed, whereas three small-scale fluctuations were confirmed. From 1996 onward, the oscillating patterns of E-30 presented a complicated phenomenon. With the increase in genetic diversity, the outbreak possibility of E-30 increased, as the genetic diversity provided the breeding grounds for subsequent outbreaks. For example, the genetic diversity of E-30 isolated in Taiwan of China increased remarkably in 2000, followed by a large outbreak of E-30 in Taiwan of China in 2001. Based on the evidence from our data, the E-30 strains isolated from mainland of China possibly originated from Taiwan of China through population movement in last century. The coastal provinces, such as Zhejiang, Fujian, and Shandong, were primary regions infected, followed by spreading to the nationwide regions.

It is important to mention here that the results of this study, which is based on the molecular epidemiological data currently available, including the *VP1* genome sequences of China, could change slightly in the future when more information will become available. The perfect HFMD pathogen surveillance system was significant for preventing infections, as was supported by the relative genetic diversity reduction after the surveillance net was built. The E-30 surveillance system has not been built yet in several countries of the world, which hinders the formulation of countermeasures for E-30 associated with encephalitis outbreaks. The basic research efforts and current surveillance should be strengthened to help understand and develop effective medical countermeasures (Gao, 2018).

## Data Availability Statement

The datasets generated for this study can be found in the GenBank database, MN590241–MN590272.

## Ethics Statement

The studies involving human participants were reviewed and approved by the Second Ethics Review Committee of the National Institute for Viral Diseases Control and Prevention, Chinese Center for Diseases Control and Prevention. Written informed consent to participate in this study was provided by the participants’ legal guardian/next of kin.

## Author Contributions

DY and YZ conceived and designed the experiments. JC and ZH performed the experiments. ZH, HW, and WX analyzed the data. JC and ZH wrote the main manuscript. ZH prepared all the tables and figures. All authors reviewed the manuscript.

## Conflict of Interest

The authors declare that the research was conducted in the absence of any commercial or financial relationships that could be construed as a potential conflict of interest.
