# Supplementary material for: A Large-Scale Outbreak of Echovirus 30 in Gansu Province of China in 2015 and Its Phylodynamic Characterization
Source: Front Microbiol. 2020 Jun 10;11:1137. doi: 10.3389/fmicb.2020.01137 (PMC7297909; doi:10.3389/fmicb.2020.01137)
Supplement: Supplementary file 1 [file Data_Sheet_1.PDF]

SUPPLEMENTARY MATERIAL

Figure S1. The result of date-randomization tests (DRTs). The temporal signal of E-30 datasets was tested using the Tip Dating Beast package.

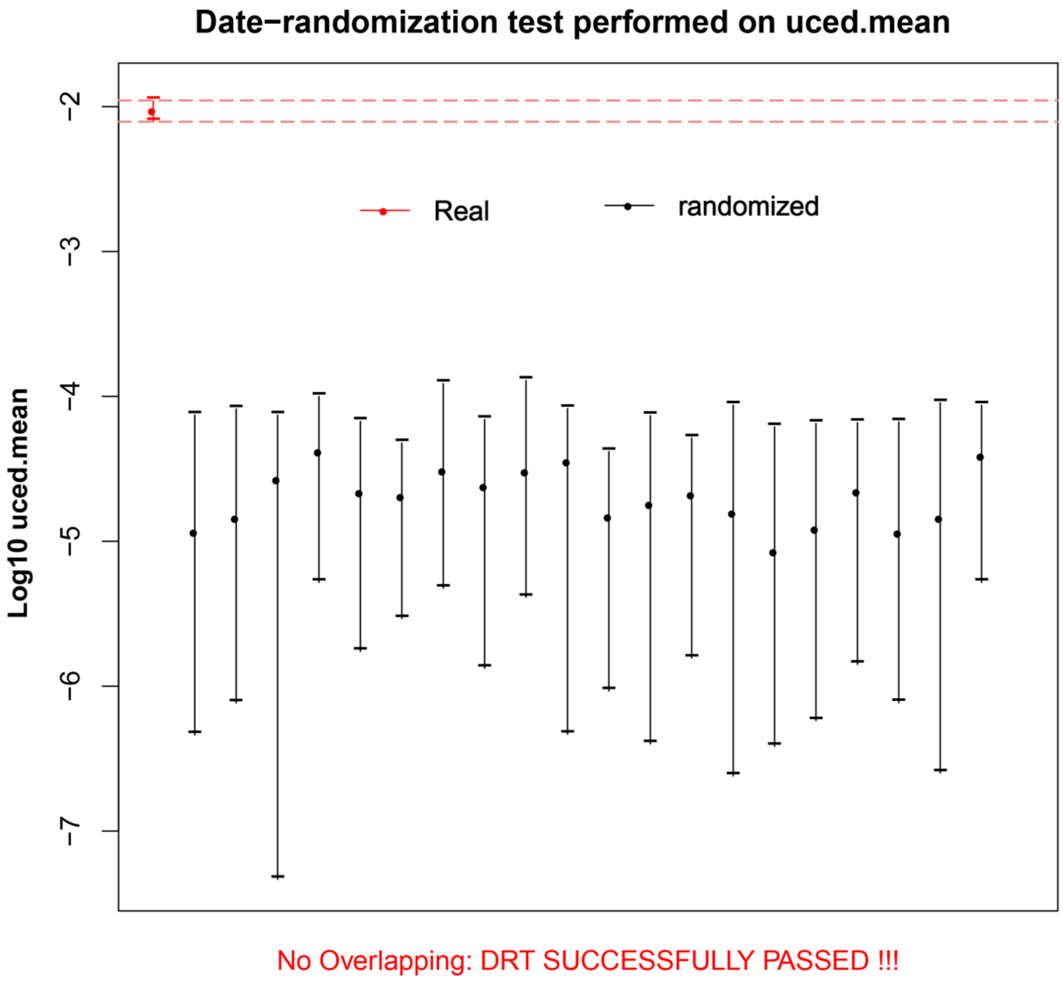

Figure S2. The model selection of BEAST. According parameters were listed, including the path sampling (PS) and stepping stone sampling (SS) values.

Data sets name	Molecular clock model	Coalescent tree prior	ESS	PS	SS
STRICT-GMRF.xml	strict clock	GMRF	>200	-17626.33992	-17637.29591
STRICT-CONSTANT.xml	strict clock	constant size	>200	-17659.95666	-17669.84234
STRICT-EXPONENTIAL.xml	strict clock	expenontial growth	>200	-17660.50927	-17672.67094
STRICT-SKYLINE.xm	strict clock	BSP	<200	-17539.87289	-17515.76623
STRICT-EBSP.xml	strict clock	EBSP	<200	N/A	N/A
E-GMRF.xml	Uncorrelated exponential relaxed clock	GMRF	>200	-17448.50542	-17466.80485
E-CONSTANT.xml	Uncorrelated exponential relaxed clock	constant size	<200	-17437.05905	-17450.37184
E-EXPONENTIAL.xml	Uncorrelated exponential relaxed clock	expenontial growth	<200	-17432.70506	-17443.08419
E-SKYLINE.xml	Uncorrelated exponential relaxed clock	BSP	<200	-17368.72737	-17381.28471
E-EBSP.xml	Uncorrelated exponential relaxed clock	EBSP	<200	N/A	N/A
L-GMRF.xml	Uncorrelated lognormal relaxed clock	GMRF	>200	-17440.79902	-17454.93468
L-CONSTANT.xml	Uncorrelated lognormal relaxed clock	constant size	>200	-17466.15909	-17477.58458
L-EXPONENTIAL.xml	Uncorrelated lognormal relaxed clock	expenontial growth	<200	-17463.01684	-17474.66929
L-SKYLINE.xml	Uncorrelated lognormal relaxed clock	BSP	<200	-17388.97137	-17403.40098
L-EBSP.xml	Uncorrelated lognormal relaxed clock	EBSP	<200	N/A	N/A
L-GMR-empty.xml	Uncorrelated lognormal relaxed clock	GMRF	<200	56403.07451	56398.56733

Table S1. The information of 460 E-30 strains used in this analysis, including 32 isolates firstly reported in this study and 14 isolates used for genotyping.

GenBank accession No.	Strain name	Countries or regions	Isolation year	Origin
MN590241	13FH-ECHO30	China	2015.49	this study
MN590242	15FR-ECHO30	China	2015.49	this study
MN590259	42YH-ECHO30	China	2015.5	this study
MN590261	44YR-ECHO30	China	2015.5	this study
MN590271	78FR-ECHO30	China	2015.5	this study
MN590243	21FH-ECHO30	China	2015.51	this study
MN590244	24FH-ECHO30	China	2015.51	this study
MN590245	26YR-ECHO30	China	2015.51	this study
MN590246	27YR-ECHO30	China	2015.51	this study
MN590247	28YR-ECHO30	China	2015.51	this study
MN590248	30YR-ECHO30	China	2015.51	this study
MN590249	31YR-ECHO30	China	2015.51	this study
MN590250	32YR-ECHO30	China	2015.51	this study
MN590251	33YR-ECHO30	China	2015.51	this study
MN590252	34YR-ECHO30	China	2015.51	this study
MN590253	35YR-ECHO30	China	2015.51	this study

MN590254	36YR-ECHO30	China	2015.51	this study
MN590255	37YR-ECHO30	China	2015.51	this study
MN590256	38YR-ECHO30	China	2015.51	this study
MN590257	39YR-ECHO30	China	2015.51	this study
MN590258	40YR-ECHO30	China	2015.51	this study
MN590260	43YR-ECHO30	China	2015.51	this study
MN590262	45YR-ECHO30	China	2015.51	this study
MN590263	53NH-ECHO30	China	2015.51	this study
MN590264	54NH-ECHO30	China	2015.51	this study
MN590265	56NH-ECHO30	China	2015.51	this study
MN590266	58NH-ECHO30	China	2015.51	this study
MN590269	74FR-ECHO30	China	2015.51	this study
MN590272	9YR-ECHO30	China	2015.51	this study
MN590270	77FR-ECHO30	China	2015.54	this study
MN590267	69FH-ECHO30	China	2015.56	this study
MN590268	72FH-ECHO30	China	2015.56	this study
AB917085.1	517-YN-HC-2013	China	2013	GenBank
AB917086.1	539-YN-HC-2013	China	2013	GenBank
AB917087.1	540-YN-HC-2013	China	2013	GenBank
AY146069.1	N1788-TW-01	China	2001	GenBank

AY146070.1	N2144-TW-01	China	2001	GenBank
AY146071.1	N2846-TW-01	China	2001	GenBank
AY146072.1	N1006-TW-01	China	2001	GenBank
AY146073.1	N2970-TW-01	China	2001	GenBank
AY146074.1	N4111-TW-00	China	2000	GenBank
AY146075.1	N2730-TW-01	China	2001	GenBank
AY146076.1	N3108-TW-01	China	2001	GenBank
AY146077.1	N1591-TW-01	China	2001	GenBank
AY146078.1	N2884-TW-01	China	2001	GenBank
AY146079.1	N1938-TW-01	China	2001	GenBank
AY146080.1	N2401-TW-01	China	2001	GenBank
AY146081.1	N0566-TW-01	China	2001	GenBank
AY146082.1	N1251-TW-01	China	2001	GenBank
AY146083.1	N1375-TW-01	China	2001	GenBank
AY146084.1	N2252-TW-01	China	2001	GenBank
AY665606.1	FD-JS03-18	China	2003	GenBank
AY665607.1	FD-JS03-30	China	2003	GenBank
AY665608.1	FD-JS03-73	China	2003	GenBank
AY665609.1	FD-JS03-102	China	2003	GenBank
AY695091.1	ECHO30/Zhejiang/6/04(CSF)	China	2004	GenBank

AY695092.1	E30/Zhejiang/28/02	China	2002	GenBank
AY695093.1	E30/Zhejiang/24/02	China	2002	GenBank
AY695094.1	Echo30/Zhejiang/12/02	China	2002	GenBank
AY695095.1	Echo30/Zhejiang/5/02	China	2002	GenBank
AY695096.1	Echo30/Zhejiang/9/02	China	2002	GenBank
AY695097.1	Echo30/Zhejiang/15/03(CSF)	China	2003	GenBank
AY695098.1	Echo30/Zhejiang/17/03(CSF)	China	2003	GenBank
AY695099.1	Echo30/Zhejiang/6/03	China	2003	GenBank
AY695100.1	Echo30/Zhejiang/2/03	China	2003	GenBank
AY695101.1	Echo30/Zhejiang/1/03(CSF)	China	2003	GenBank
AY695102.1	Echo30/Zhejiang/3/03	China	2003	GenBank
AY695103.1	Echo30/Zhejiang/3/04(CSF)	China	2004	GenBank
AY695104.1	Echo30/Zhejiang/11/04	China	2004	GenBank
AY695105.1	Echo30/Zhejiang/5/04(CFS)	China	2004	GenBank
AY695106.1	Echo30/Zhejiang/23/02(CFS)	China	2002	GenBank
AY695107.1	Echo30/Zhejiang/10/04(CFS)	China	2004	GenBank
AY714345.1	Echo30/zhejiang/4/04(CSF)	China	2004	GenBank
AY714346.1	Echo30/zhejiang/1/04	China	2004	GenBank
AY879315.1	Echo30/Zhejiang/2/04(CSF)	China	2004	GenBank
AY879316.1	Echo30/Zhejiang/17/04(CSF)	China	2004	GenBank

AY879317.1	Echo30/Zhejiang/23/04(CSF)	China	2004	GenBank
AY879318.1	Echo30/Zhejiang/39/04(CSF)	China	2004	GenBank
AY879319.1	Echo30/Zhejiang/41/04(CSF)	China	2004	GenBank
AY879320.1	Echo30/Zhejiang/50/04(CSF)	China	2004	GenBank
AY879321.1	Echo30/Zhejiang/63/04(CSF)	China	2004	GenBank
AY879322.1	Echo30/Zhejiang/66/04(CSF)	China	2004	GenBank
AY879323.1	Echo30/Zhejiang/87/04(CSF)	China	2004	GenBank
AY879324.1	Echo30/Zhejiang/100/04(CSF)	China	2004	GenBank
AY879325.1	Echo30/Zhejiang/101/04(CSF)	China	2004	GenBank
AY879326.1	Echo30/Zhejiang/113/04(CSF)	China	2004	GenBank
DQ118682.1	SD03-ta-70	China	2003	GenBank
DQ118683.1	SD03-TA-3	China	2003	GenBank
DQ118684.1	SD03-TA-11	China	2003	GenBank
DQ118685.1	SD03-ZQ-08	China	2003	GenBank
DQ118686.1	SD03-ZQ-11	China	2003	GenBank
DQ118687.1	SD03-ZQ-10	China	2003	GenBank
DQ118688.1	SD03-ZQ-14	China	2003	GenBank
DQ118689.1	SD03-ZQ-20	China	2003	GenBank
DQ118690.1	SD03-ZQ-21	China	2003	GenBank
DQ118691.1	SD03-ZQ-24	China	2003	GenBank

DQ118692.1	SD03-ZQ-26	China	2003	GenBank
DQ118693.1	SD03-ZQ-25	China	2003	GenBank
DQ118694.1	SD03-ZQ-29	China	2003	GenBank
DQ118695.1	SD03-ZQ-33	China	2003	GenBank
DQ118696.1	SD03-ZQ-35	China	2003	GenBank
DQ118697.1	SD03-ZQ-44	China	2003	GenBank
DQ118698.1	SD03-ZQ-47	China	2003	GenBank
DQ118699.1	SD03-ZQ-50	China	2003	GenBank
DQ118700.1	SD03-ZQ-51	China	2003	GenBank
DQ205335.1	SD03-ZQ-02	China	2003	GenBank
DQ205336.1	SD03-ZQ-07	China	2003	GenBank
DQ205337.1	SD03-ZQ-09	China	2003	GenBank
DQ246620.1	Echo30/Zhejiang/17/03/CSF	China	2003	GenBank
DQ842242.1	2813-05-TW	China	2005	GenBank
DQ842243.1	384-88-TW	China	1988	GenBank
DQ842244.1	517-88-TW	China	1988	GenBank
DQ842245.1	552-88-TW	China	1988	GenBank
DQ842246.1	692-88-TW	China	1988	GenBank
DQ842247.1	726-88-TW	China	1988	GenBank
DQ842248.1	837-88-TW	China	1988	GenBank

DQ842249.1	269-89-TW	China	1989	GenBank
DQ842250.1	026-93-TW	China	1993	GenBank
DQ842251.1	104-93-TW	China	1993	GenBank
DQ842252.1	294-93-TW	China	1993	GenBank
DQ842253.1	310-93-TW	China	1993	GenBank
DQ842254.1	335-93-TW	China	1993	GenBank
DQ842255.1	337-93-TW	China	1993	GenBank
DQ842256.1	1301-93-TW	China	1993	GenBank
DQ842257.1	4714-00-TW	China	2000	GenBank
DQ842258.1	117-01-TW	China	2001	GenBank
DQ842259.1	681-01-TW	China	2001	GenBank
DQ842260.1	1794-01-TW	China	2001	GenBank
DQ842261.1	N0566-01-TW	China	2001	GenBank
DQ842262.1	N1788-01-TW	China	2001	GenBank
DQ842263.1	N2280-01-TW	China	2001	GenBank
DQ842264.1	N3108-01-TW	China	2001	GenBank
DQ842265.1	N4111-01-TW	China	2001	GenBank
EF066391.1	TW/2513/01	China	2001	GenBank
EF066392.1	TW/3182/01	China	2001	GenBank
EF596823.1	267-89-TW	China	1989	GenBank

EF596824.1	312-01-TW	China	2001	GenBank
EF596825.1	2210-01-TW	China	2001	GenBank
EF596826.1	N0094-01-TW	China	2001	GenBank
FJ919565.1	017/2008TC/SD/CHN	China	2008.53	GenBank
GQ329836.1	05239/SD/CHN/2005/E30	China	2005	GenBank
GQ329837.1	06530/SD/CHN/2006/E30	China	2006	GenBank
GQ329838.1	AM37010407071/SD/CHN/2007/E30	China	2007	GenBank
HQ436345.1	GX10/05	China	2010	GenBank
HQ436346.1	GX10/08	China	2010	GenBank
HQ436347.1	GX10/15	China	2010	GenBank
HQ625643.1	Henan/01/2008	China	2008.57	GenBank
HQ625644.1	Henan/02/2008	China	2008.58	GenBank
HQ625645.1	Henan/03/2008	China	2008.57	GenBank
HQ625646.1	Henan/04/2008	China	2008.52	GenBank
HQ625647.1	Henan/05/2008	China	2008.56	GenBank
HQ625648.1	Henan/06/2008	China	2008.54	GenBank
HQ625649.1	Henan/07/2008	China	2008.57	GenBank
HQ625650.1	Henan/08/2008	China	2008.65	GenBank
JF823636.1	E30.2010Linyi.005ST	China	2010	GenBank
JF823637.1	E30.2010Linyi.011CSF/T	China	2010	GenBank

JF823638.1	E30.2010Linyi.014CSF	China	2010	GenBank
JF823639.1	E30.2010Linyi.040T/ST	China	2010	GenBank
JF823640.1	E30.2010Linyi.060T	China	2010	GenBank
JF823641.1	E30.2010Linyi.068T	China	2010	GenBank
JF823642.1	E30.2010Linyi.069CSF	China	2010	GenBank
JF823643.1	E30.2010Linyi.075T	China	2010	GenBank
JF823644.1	E30.2010Linyi.077CSF	China	2010	GenBank
JF823645.1	E30.2010Linyi.086ST	China	2010	GenBank
JF823646.1	E30.2010Linyi.092ST	China	2010	GenBank
JX028208.1	ZJ/WZ-35/08	China	2008.04	GenBank
JX028209.1	ZJ/WZ-161/08	China	2008.78	GenBank
JX028210.1	ZJ/WZ-191/08	China	2008.78	GenBank
JX028211.1	ZJ/WZ-1/09	China	2009.04	GenBank
JX028212.1	ZJ/QZ-48/09	China	2009.37	GenBank
JX028213.1	ZJ/QZ-37/10	China	2010.37	GenBank
JX028214.1	ZJ/QZ-39/10	China	2010.37	GenBank
JX028215.1	ZJ/QZ-54/10	China	2010.45	GenBank
JX028216.1	ZJ/QZ-65/10	China	2010.45	GenBank
JX028217.1	ZJ/WZ-8/11	China	2011.53	GenBank
JX028218.1	ZJ/WZ-13/11	China	2011.53	GenBank

JX028219.1	ZJ/WZ-14/11	China	2011.53	GenBank
JX028220.1	ZJ/WZ-15/11	China	2011.53	GenBank
JX028221.1	ZJ/WZ-18/11	China	2011.53	GenBank
JX028222.1	ZJ/WZ-28/11	China	2011.62	GenBank
JX028223.1	ZJ/WZ-30/11	China	2011.62	GenBank
JX129810.1	CSF11-19	China	2011	GenBank
JX129811.1	CSF11-1	China	2011	GenBank
JX129812.1	CSF11-18	China	2011	GenBank
JX129813.1	CSF11-161	China	2011	GenBank
JX129814.1	CSF11-5	China	2011	GenBank
JX129815.1	ACSF11-7	China	2011	GenBank
JX129816.1	FCSF11-15	China	2011	GenBank
JX129817.1	FCSF11-22	China	2011	GenBank
JX129818.1	CSF11-179	China	2011	GenBank
JX129819.1	CSF11-58	China	2011	GenBank
JX129820.1	FCSF11-14	China	2011	GenBank
JX129821.1	FCSF11-23	China	2011	GenBank
JX129822.1	CSF11-24	China	2011	GenBank
JX129823.1	CSF11-21	China	2011	GenBank
JX129824.1	CSF11-49	China	2011	GenBank

JX129825.1	VE11-4TS	China	2011	GenBank
JX129826.1	VE11-7TS	China	2011	GenBank
JX129827.1	VE11-11TS	China	2011	GenBank
JX129828.1	CSF11-68	China	2011	GenBank
JX129829.1	CSF11-47	China	2011	GenBank
JX129830.1	CSF11-45	China	2011	GenBank
JX129831.1	CSF11-51	China	2011	GenBank
JX129832.1	CSF11-46	China	2011	GenBank
JX129833.1	CSF11-165	China	2011	GenBank
JX129834.1	CSF05-63	China	2005	GenBank
JX129835.1	CSF05-92	China	2005	GenBank
JX129836.1	CSF06-34	China	2006	GenBank
JX129837.1	AFP05-53	China	2005	GenBank
JX129838.1	CSF06-7	China	2005	GenBank
JX129839.1	CSF06-9	China	2005	GenBank
JX129840.1	CSF04-38	China	2004	GenBank
JX129841.1	CSF05-21	China	2005	GenBank
JX129842.1	CSF05-75	China	2005	GenBank
JX129843.1	CSF09-79	China	2009	GenBank
JX129844.1	CSF09-95	China	2009	GenBank

JX129845.1	CSF09-106	China	2009	GenBank
JX129846.1	CSF09-63	China	2009	GenBank
JX129847.1	CSF09-85	China	2009	GenBank
JX129848.1	AFP08-104	China	2008	GenBank
JX129849.1	CSF08-141	China	2008	GenBank
JX129850.1	VE11-19F	China	2011	GenBank
JX129851.1	VE11-18F	China	2011	GenBank
JX129852.1	VE11-17TS	China	2011	GenBank
JX129853.1	CSF11-170	China	2011	GenBank
JX129854.1	ACSF11-13	China	2011	GenBank
JX129855.1	CSF11-107	China	2011	GenBank
JX129856.1	CSF11-115	China	2011	GenBank
JX129857.1	CSF11-112	China	2011	GenBank
JX129858.1	CSF11-109	China	2011	GenBank
JX129859.1	CSF11-114	China	2011	GenBank
JX129860.1	CSF11-64	China	2011	GenBank
JX129861.1	CSF11-86	China	2011	GenBank
JX129862.1	CSF11-39	China	2011	GenBank
JX129863.1	CSF11-73	China	2011	GenBank
JX129864.1	CSF11-50	China	2011	GenBank

JX129865.1	CSF11-130	China	2011	GenBank
JX129866.1	CSF11-191	China	2011	GenBank
JX129867.1	CSF11-190	China	2011	GenBank
JX129868.1	CSF11-129	China	2011	GenBank
JX129869.1	CSF11-59	China	2011	GenBank
JX129870.1	CSF11-104	China	2011	GenBank
JX129871.1	CSF11-158	China	2011	GenBank
JX129872.1	CSF11-75	China	2011	GenBank
JX129873.1	AFP01-56	China	2001	GenBank
JX129874.1	AFP04-138	China	2004	GenBank
JX129875.1	CSF05-27	China	2005	GenBank
JX129876.1	CSF2004-C2	China	2004	GenBank
JX129877.1	CSF2004-C7	China	2004	GenBank
JX129878.1	CSF05-33	China	2005	GenBank
JX129879.1	CSF05-25	China	2005	GenBank
JX129880.1	E05-5	China	2005	GenBank
JX129881.1	CSF05-30	China	2005	GenBank
JX854435.1	ECV30/GX10/05	China	2010	GenBank
JX976773.1	E30SD2010CHN	China	2010.53	GenBank
KC867101.1	JB14080452	China	2008.37	GenBank

KC867102.1	JB141230267	China	2012.62	GenBank
KC897073.1	2012EM161	China	2012.44	GenBank
KF015730.1	A336/KM/2009	China	2009.38	GenBank
KF246764.1	100/JN/CHN/AM/06/E30	China	2010	GenBank
KF246765.1	191/JN/CHN/AM/08/E30	China	2010	GenBank
KF246766.1	002/LS/CHN/AM/08/E30	China	2010	GenBank
KF246767.1	006/LS/CHN/AM/08/E30	China	2010	GenBank
KF246768.1	036/CS/CHN/AM/09/E30	China	2010	GenBank
KF246769.1	LU/JN/CHN/AM/10/E30	China	2010	GenBank
KF246770.1	ZYH/JN/CHN/AM/10/E30	China	2010	GenBank
KF246771.1	015/JN/CHN/AM/11/E30	China	2010	GenBank
KF246772.1	LQZ/JN/CHN/AM/11/E30	China	2010	GenBank
KF246773.1	JE026/SD/CHN/10/E30	China	2010	GenBank
KF246774.1	JE004/SD/CHN/12/E30	China	2010	GenBank
KF878942.1	KM/A363/09	China	2009.98	GenBank
KM034781.1	C1/GD/CHN/2012	China	2012.38	GenBank
KM034782.1	C3/GD/CHN/2012	China	2012.36	GenBank
KM034783.1	C8/GD/CHN/2012	China	2012.36	GenBank
KM034784.1	C11/GD/CHN/2012	China	2012.36	GenBank
KM034785.1	C13/GD/CHN/2012	China	2012.38	GenBank

KM034786.1	C14/GD/CHN/2012	China	2012.36	GenBank
KM034787.1	C15/GD/CHN/2012	China	2012.36	GenBank
KM034788.1	C16/GD/CHN/2012	China	2012.35	GenBank
KM034789.1	C17/GD/CHN/2012	China	2012.38	GenBank
KM034790.1	C19/GD/CHN/2012	China	2012.36	GenBank
KM034791.1	C20/GD/CHN/2012	China	2012.37	GenBank
KM034792.1	C21/GD/CHN/2012	China	2012.37	GenBank
KM034793.1	C22/GD/CHN/2012	China	2012.37	GenBank
KM034794.1	C23/GD/CHN/2012	China	2012.37	GenBank
KM034795.1	C24/GD/CHN/2012	China	2012.36	GenBank
KM034796.1	C25/GD/CHN/2012	China	2012.36	GenBank
KM034797.1	C26/GD/CHN/2012	China	2012.36	GenBank
KM034798.1	C29/GD/CHN/2012	China	2012.38	GenBank
KM034799.1	C33/GD/CHN/2012	China	2012.38	GenBank
KM034800.1	21-1-3/GD/CHN/2012	China	2012.41	GenBank
KM034801.1	23-1-3/GD/CHN/2012	China	2012.41	GenBank
KM034802.1	23-1-4/GD/CHN/2012	China	2012.41	GenBank
KM034803.1	24-1-2/GD/CHN/2012	China	2012.41	GenBank
KM034804.1	50-1-2/GD/CHN/2010	China	2010.62	GenBank
KM034805.1	41-1-1/GD/CHN/2011	China	2011.79	GenBank

KM034806.1	32-2-1/GD/CHN/2012	China	2012.52	GenBank
KP266571.1	2002-59	China	2002.37	GenBank
KP985769.1	ZJ/RA-72/13	China	2013.56	GenBank
KP985770.1	ZJ/LC-94/13	China	2013.32	GenBank
KP985771.1	ZJ/LC-9/14	China	2014.63	GenBank
KP985772.1	ZJ/JH-Chen/14	China	2014.79	GenBank
KP985773.1	ZJ/JH-Li/14	China	2014.79	GenBank
KP985774.1	ZJ/JH-Hu/14	China	2014.79	GenBank
KP985775.1	ZJ/ZY-2/14	China	2014.54	GenBank
KP985779.1	ZJ/RA-3/13	China	2013.87	GenBank
KP985780.1	ZJ/LC-26/13	China	2013.48	GenBank
KP985781.1	ZJ/LC-108/13	China	2013.63	GenBank
KR231964.1	HZ-1-E30/ZJ/2003	China	2003	GenBank
KR231965.1	YK-4-E30/ZJ/2004	China	2004	GenBank
KR231966.1	PT-113-E30/ZJ/2004	China	2004	GenBank
KR231967.1	YQ-2-E30/ZJ/2004	China	2004	GenBank
KR231968.1	LH-39-E30/ZJ/2004	China	2004	GenBank
KR231969.1	LH-41-E30/ZJ/2004	China	2004	GenBank
KR231972.1	HY-8-E30/ZJ/2012	China	2012	GenBank
KR231973.1	HY-3-E30/ZJ/2012	China	2012	GenBank

KR231974.1	HY-1-E30/ZJ/2012	China	2012	GenBank
KR231975.1	HY-6-E30/ZJ/2012	China	2012	GenBank
KR231977.1	M-16-E30/ZJ/2012	China	2012	GenBank
KR231979.1	PT-2-E30/ZJ/2012	China	2012	GenBank
KR231980.1	PT-3-E30/ZJ/2012	China	2012	GenBank
KR231981.1	PT-5-E30/ZJ/2012	China	2012	GenBank
KR231982.1	PT-6-E30/ZJ/2012	China	2012	GenBank
KR231986.1	RA-124-E30/ZJ/2012	China	2012	GenBank
KR231987.1	RA-138-E30/ZJ/2012	China	2012	GenBank
KR231988.1	RA-149-E30/ZJ/2012	China	2012	GenBank
KR231989.1	RA-150-E30/ZJ/2012	China	2012	GenBank
KR231990.1	RA-151-E30/ZJ/2012	China	2012	GenBank
KR231992.1	RA-32-E30/ZJ/2012	China	2012	GenBank
KR231997.1	RA-42-E30/ZJ/2012	China	2012	GenBank
KR231998.1	RA-43-E30/ZJ/2012	China	2012	GenBank
KR232000.1	RA-5-E30/ZJ/2012	China	2012	GenBank
KR232001.1	RA-50-E30/ZJ/2012	China	2012	GenBank
KR232002.1	RA-52-E30/ZJ/2012	China	2012	GenBank
KR232005.1	RA-12-61-E30/ZJ/2012	China	2012	GenBank
KR232006.1	RA-62-E30/ZJ/2012	China	2012	GenBank

KR232010.1	RA-76-E30/ZJ/2012	China	2012	GenBank
KR232012.1	RA-79-E30/ZJ/2012	China	2012	GenBank
KR232013.1	RA-85-E30/ZJ/2012	China	2012	GenBank
KR232016.1	ZS-2-E30/ZJ/2012	China	2012	GenBank
KR232017.1	ZS-3-E30/ZJ/2012	China	2012	GenBank
KR232018.1	ZS-4-E30/ZJ/2012	China	2012	GenBank
KR232019.1	ZS-5-E30/ZJ/2012	China	2012	GenBank
KR232020.1	ZS-6-E30/ZJ/2012	China	2012	GenBank
KT353720.1	1-B4-TW	China	2008	GenBank
KT633561.1	29/Kaihua/Zhejiang/CHN/2012/E30	China	2012.7	GenBank
KU665301.1	E30/A118/YN/CHN/2009	China	2009.55	GenBank
KU665302.1	E30/F53/YN/CHN/2010	China	2010.12	GenBank
KU665303.1	E30/F115/YN/CHN/2010	China	2010.25	GenBank
KU665304.1	E30/F8/YN/CHN/2010	China	2010.01	GenBank
KU665305.1	E30/F5/YN/CHN/2010	China	2010.1	GenBank
KU665306.1	E30/F42/YN/CHN/2010	China	2010.55	GenBank
KU665307.1	E30/F44/YN/CHN/2010	China	2010.55	GenBank
KU665308.1	E30/F134/YN/CHN/2010	China	2010.56	GenBank
KU665309.1	E30/F97/YN/CHN/2010	China	2010.56	GenBank
KU665310.1	E30/A180/YN/CHN/2009	China	2009.56	GenBank

KU665311.1	E30/A203/YN/CHN/2009	China	2009.58	GenBank
KU665312.1	E30/A277/YN/CHN/2009	China	2009.61	GenBank
KU665313.1	E30/A283/YN/CHN/2009	China	2009.64	GenBank
KU665314.1	E30/A283/YN/CHN/2009	China	2009.64	GenBank
KX261884.1	DT-F-9	China	2015.75	GenBank
KX261885.1	DT-F-18	China	2015.82	GenBank
KX261886.1	DT-Y-17	China	2015.75	GenBank
KX261887.1	DT-Y-9	China	2015.87	GenBank
KX687812.1	16JJ01-2016.5	China	2016.37	GenBank
KX687813.1	16JJ02-2016.5	China	2016.37	GenBank
KX687814.1	16JJ04-2016.5	China	2016.37	GenBank
KX687815.1	16JJ05-2016.5	China	2016.37	GenBank
KX687816.1	16JJ07-2016.5	China	2016.37	GenBank
KX687817.1	16JJ09-2016.5	China	2016.37	GenBank
KX687818.1	16JJ10-2016.5	China	2016.37	GenBank
KX687819.1	16JJ11-2016.5	China	2016.37	GenBank
KX687820.1	16JJ29-2016.5	China	2016.37	GenBank
KX687821.1	16JJ33-2016.5	China	2016.37	GenBank
KX687822.1	16JJ40-2016.5	China	2016.37	GenBank
KY048010.1	C10/SD/CHN/AM/14	China	2014	GenBank

KY048011.1	C13/SD/CHN/AM/14	China	2014	GenBank
KY048012.1	C14/SD/CHN/AM/14	China	2014	GenBank
KY048013.1	C16/SD/CHN/AM/14	China	2014	GenBank
KY048014.1	C17/SD/CHN/AM/14	China	2014	GenBank
KY048015.1	C18/SD/CHN/AM/14	China	2014	GenBank
KY048016.1	C24/SD/CHN/AM/14	China	2014	GenBank
KY048017.1	C25/SD/CHN/AM/14	China	2014	GenBank
KY048018.1	C43/SD/CHN/AM/14	China	2014	GenBank
KY048019.1	C46/SD/CHN/AM/14	China	2014	GenBank
KY048020.1	C47/SD/CHN/AM/14	China	2014	GenBank
KY048021.1	C117/SD/CHN/AM/14	China	2014	GenBank
KY048022.1	C461/SD/CHN/AM/14	China	2014	GenBank
KY048023.1	C463/SD/CHN/AM/14	China	2014	GenBank
KY048024.1	C469/SD/CHN/AM/14	China	2014	GenBank
KY048025.1	C470/SD/CHN/AM/14	China	2014	GenBank
KY048026.1	C472/SD/CHN/AM/14	China	2014	GenBank
KY048027.1	C473/SD/CHN/AM/14	China	2014	GenBank
KY048028.1	C475/SD/CHN/AM/14	China	2014	GenBank
KY048029.1	C478/SD/CHN/AM/14	China	2014	GenBank
KY048030.1	C482/SD/CHN/AM/14	China	2014	GenBank

KY048031.1	C485/SD/CHN/AM/14	China	2014	GenBank
KY048032.1	C488/SD/CHN/AM/14	China	2014	GenBank
KY048033.1	C500/SD/CHN/AM/14	China	2014	GenBank
KY048034.1	C548/SD/CHN/AM/14	China	2014	GenBank
KY048035.1	C646/SD/CHN/AM/14	China	2014	GenBank
KY048036.1	C660/SD/CHN/AM/14	China	2014	GenBank
KY048037.1	C694/SD/CHN/AM/14	China	2014	GenBank
KY048038.1	C700/SD/CHN/AM/14	China	2014	GenBank
KY048039.1	C738/SD/CHN/AM/14	China	2014	GenBank
KY048040.1	FDT/SD/CHN/AM/14	China	2014	GenBank
KY048041.1	LK03T/SD/CHN/AM/14	China	2014	GenBank
KY048042.1	LK04T/SD/CHN/AM/14	China	2014	GenBank
KY048043.1	LK06/SD/CHN/AM/14	China	2014	GenBank
KY048044.1	LKZWL/SD/CHN/AM/14	China	2014	GenBank
LC120939.1	192-YN-CHN-2015JK	China	2015	GenBank
LC120940.1	274-YN-CHN-2015JK	China	2015	GenBank
LC120941.1	287-YN-CHN-2015JK	China	2015	GenBank
LC128680.1	105B-YN-CHN-2010	China	2010	GenBank
LC128681.1	106B-YN-CHN-2010	China	2010	GenBank
LC128683.1	110B-YN-CHN-2010	China	2010	GenBank

LC128684.1	112B-YN-CHN-2010	China	2010	GenBank
LC128685.1	113B-YN-CHN-2010	China	2010	GenBank
LC128686.1	285B-YN-CHN-2011	China	2011	GenBank
LC128687.1	355B-YN-CHN-2011	China	2011	GenBank
LC128688.1	356B-YN-CHN-2011	China	2011	GenBank
LC167441.1	34-YN-CHN-2016HC	China	2016	GenBank
LC201505.1	37R-YN-CHN-2016HC	China	2016	GenBank
LC201506.1	42R-YN-CHN-2016HC	China	2016	GenBank
LC201507.1	43R-YN-CHN-2016HC	China	2016	GenBank
LC201508.1	45R-YN-CHN-2016HC	China	2016	GenBank
LC201509.1	46R-YN-CHN-2016HC	China	2016	GenBank
LC201510.1	51R-YN-CHN-2016HC	China	2016	GenBank
LC201511.1	61R-YN-CHN-2016HC	China	2016	GenBank
LC361295.1	SF113	China	2016.38	GenBank
LC361296.1	SF111	China	2016.38	GenBank
LC361297.1	SF112	China	2016.38	GenBank
LC361298.1	SF156	China	2016.38	GenBank
MF422573.1	61248-1425	China	2008	GenBank
MF422574.1	61249-244	China	2008	GenBank
MF422575.1	61250-858	China	2008	GenBank

MF422576.1	61251-440	China	2008	GenBank
MF422577.1	63037-1463	China	2008	GenBank
MF422578.1	63039-1999	China	2008	GenBank
AY371481.1	17891-BYE02	Byelorussia	2002	GenBank
AJ241451.1	97CF551	France	1997	GenBank
AM711029.1	302024-05	France	2005	GenBank
HG793748.1	CF168036/CSF-4.74-FRA09	France	2009	GenBank
HG793778.1	CF222040/CSF-4.62-FRA09	France	2009	GenBank
AF236538.1	22isr98	Israel	1998	GenBank
JN704615.1	Kor08-ECV30	Korea	2008.53	GenBank
EU293776.1	SB6337	Malaysia	2001	GenBank
AF236532.1	7821net93	Netherlands	1993	GenBank
KP090704.1	27635-BEL-2006	Russia	2006	GenBank
KP090708.1	28417-RUS-2007	Russia	2007	GenBank
KP090760.1	3805-RU-MOW-2010	Russia	2010	GenBank
KP261897.1	E30-4157/Penza/RU/2013	Russia	2013	GenBank
KP261901.1	E30-2431/Tambov/RU/2013	Russia	2013	GenBank
JN177727.1	Es6-01.TUN1992	Tunisia	1992	GenBank
JN177729.1	E125-01.TUN2001	Tunisia	2001	GenBank
AF128014.1	ma91-0929	USA	1991	GenBank

AF128045.1	nc79-1305	USA	1979	GenBank
AF128053.1	pa81-3399	USA	1981	GenBank
AF162711.1	Bastianni	USA	1958	GenBank
AY302547	Farina	USA	1950	GenBank
LC361295.1	SF113	China	2016.38	GenBank
LC361296.1	SF111	China	2016.38	GenBank
LC361297.1	SF112	China	2016.38	GenBank
LC361298.1	SF156	China	2016.38	GenBank
MF422573.1	61248-1425	China	2008	GenBank
MF422574.1	61249-244	China	2008	GenBank
MF422575.1	61250-858	China	2008	GenBank
MF422576.1	61251-440	China	2008	GenBank
MF422577.1	63037-1463	China	2008	GenBank
MF422578.1	63039-1999	China	2008	GenBank
AY371481.1	17891-BYE02	Byelorussia	2002	GenBank
AJ241451.1	97CF551	France	1997	GenBank
AM711029.1	302024-05	France	2005	GenBank
HG793748.1	CF168036/CSF-4.74-FRA09	France	2009	GenBank
HG793778.1	CF222040/CSF-4.62-FRA09	France	2009	GenBank
AF236538.1	22isr98	Israel	1998	GenBank

JN704615.1	Kor08-ECV30	Korea	2008.53	GenBank
EU293776.1	SB6337	Malaysia	2001	GenBank
AF236532.1	7821net93	Netherlands	1993	GenBank
KP090704.1	27635-BEL-2006	Russia	2006	GenBank
KP090708.1	28417-RUS-2007	Russia	2007	GenBank
KP090760.1	3805-RU-MOW-2010	Russia	2010	GenBank
KP261897.1	E30-4157/Penza/RU/2013	Russia	2013	GenBank
KP261901.1	E30-2431/Tambov/RU/2013	Russia	2013	GenBank
JN177727.1	Es6-01.TUN1992	Tunisia	1992	GenBank
JN177729.1	E125-01.TUN2001	Tunisia	2001	GenBank
AF128014.1	ma91-0929	USA	1991	GenBank
AF128045.1	nc79-1305	USA	1979	GenBank
AF128053.1	pa81-3399	USA	1981	GenBank
AF162711.1	Bastianni	USA	1958	GenBank
AY302547	Farina	USA	1950	GenBank
